# “To make a positive difference”: diverse youth perspectives on getting involved in mental health research

**DOI:** 10.1186/s40900-026-00882-9

**Published:** 2026-05-18

**Authors:** Rachel Perowne, Sarah Rowe, Azin Lajevardi, Leslie Morrison Gutman

**Affiliations:** 1 Division of Psychology and Language Sciences, UCL Centre for Behaviour Change, University College London, London, UK; 2 Division of Psychiatry, University College London, London, UK; 3 Bristol, UK

**Keywords:** Youth, Mental health, Patient and public involvement, Diverse, Under-represented

## Abstract

**Background:**

Mental health problems amongst young people in the UK have been rising, yet many still do not receive appropriate support. Involving young people in research can lead to more inclusive services. However, certain groups of young people, including those from ethnic minority backgrounds, lower-income families, refugee, asylum-seeking and other immigrant backgrounds, non-native English speakers, disabled young people and those with long term health conditions remain under-represented in involvement in mental health research. Little research explores the perspectives of these young people on the barriers and enablers to their involvement.

**Methods:**

This qualitative focus group study used the Behaviour Change Wheel (BCW) and Capability-Opportunity-Motivation model of behaviour (COM-B) to understand influencers on involvement. A group of youth advisors was involved throughout. Five focus groups were conducted, including 18 young people aged 16–25, many from under-represented backgrounds and without previous involvement experience. Thematic analysis was used to categorise barriers and enablers according to the COM-B model and intervention strategies proposed using intervention functions and behaviour change techniques which form part of the BCW.

**Results:**

Nineteen themes were identified covering all six COM-B domains. Enablers included the perceived value and relevance of the research topic, personal benefits (including financial reward and skill development), as well as safe and inclusive environments where young people of shared experiences come together. Barriers included lack of awareness and access to involvement opportunities, emotional readiness, social norms and the influence of peers. Some barriers were highlighted as particularly relevant to certain groups. Youth advisors provided additional reflections on the findings. BCW informed intervention strategies include proactive outreach efforts, effective preparation of young people (including induction and capacity building), the use of role models and partnership with cultural organisations.

**Conclusions:**

Many barriers to inclusive youth involvement in mental health research exist. Researchers need to look beyond traditional approaches to include under-represented young people in research. This study highlights enablers that researchers can capitalise on to improve access to involvement opportunities and BCW derived strategies, supported by evidence and young people’s ideas, to overcome barriers. Future research should implement and evaluate these strategies in practice.

**Supplementary Information:**

The online version contains supplementary material available at 10.1186/s40900-026-00882-9.

## Abstract (plain language summary)

Mental health problems amongst young people in the UK are rising but many struggle to get help. Involving young people in research can lead to better treatment and services. However, those from certain backgrounds are under-represented, meaning that services may not be suitable for them. In this study 18 young people aged 16–25, from groups whose voices are not often heard in research, attended focus groups as participants. Data was collected from them to understand the enablers (what helps) and barriers (what hinders) their involvement. A behaviour change model called the Behaviour Change Wheel, was used to analyse what participants said. This model helps researchers understand what motivates people and what skills and support they need for change to happen. Young people were involved throughout this study, as advisors as well as youth researchers, including contributing to writing the article. Overall nineteen themes were found. Young people were more likely to get involved in research when they thought the topic was worthwhile and relevant to them, where they could see personal benefits (such as being paid) and where they felt safe and welcomed in the research environment. They were put off being involved when they did not understand what being involved in research meant or where opportunities to get involved weren’t easily available to them. Another major barrier included worries about what their friends or those around them would think of their involvement. Researchers can help improve youth involvement by going to young people where they are, preparing them for involvement, working with local organisations and role models to show young people that involvement is relevant to them. The next step would be to put these ideas in place and test their effectiveness.

**Supplementary Information:**

The online version contains supplementary material available at 10.1186/s40900-026-00882-9.

## Background

Levels of reported mental health problems in young people in England have been increasing over a number of years. In 2021, 16.9% of 16–23-year-olds reported a probable mental health condition [1] compared to 23.3% of 17–19-year-olds and 21.7% of 20–25-year-olds in 2023 [2]. However, increasing demand on Children and Adolescent Mental Health Services (CAMHS), which are often seen as over-stretched, fragmented and inaccessible [3, 4], combined with a reluctance among some young people to seek help [5], leaves many young people unsupported.

Rates of mental health help-seeking vary between groups of young people, with those of certain identities being less likely to access help. For example, a recent systematic review found that stigmatisation in certain cultures and a reliance on community leaders rather than mental health professionals, in some faiths, influenced help-seeking amongst diverse university students [6]. The study identified a fear of discrimination amongst young ethnic minority men and a belief that mental health professionals lack cultural competence as barriers [6]. Other studies also report masculine norms to be a barrier to mental health help-seeking amongst adolescent boys [7] and fear of discrimination hindering lesbian, gay and bisexual young people’s help-seeking [8]. Young people in out of home care report fears of stigmatisation and lack of understanding of their circumstances as barriers to help-seeking [9].

Overcoming these challenges is essential in providing appropriate and accessible treatment for all young people. Children and young people have the right, as set out in article 12 of the Convention on the Rights of the Child, to express themselves and for their views to be given weight [10]. This includes in shared decision making, where clinicians and patients work together to make decisions about care or treatment [11], and including youth voice in the research process [12]. To achieve this, the mental health research that informs treatment and service development should include participants from a wide range of backgrounds, and particularly those who have traditionally been less well served [13]. Further, involving diverse lived-experience experts throughout the research process, not solely as study participants but as “active partners” [14], (for example through Patient and Public Involvement activities) improves representation in participant recruitment, enhances the quality and appropriateness of the research, empowers those involved and raises community awareness [15–17]. Within youth mental health research specifically, these benefits are mirrored; with positive outcomes such as improving research relevance and personal development for young people and researchers identified [12]. However, experts agree that young people from ethnic minority and immigrant backgrounds, non-native English speakers, disabled young people and those from lower-income families, are poorly represented in youth mental health research involvement in the United Kingdom [18], which risks limiting the generalisability of findings and perpetuating inequalities in health policy [19].

The literature references numerous methods and models of involvement [18, 20]. At its core, involvement means conducting research “with” rather than “to” the population being studied [21]. For young people, this might involve sharing their views and experiences as part of an advisory group, reviewing study materials or taking an active role in conducting research themselves, as a youth co-researcher [18]. There are criticisms that these existing mechanisms of involvement often rely on the ‘usual suspects’ and are not inclusive or accessible for those whose voices are less often heard [22].

A number of recent reviews of young people’s involvement in mental health research report barriers and facilitators to their engagement, including both relational and process factors [12, 20, 23]. Strong relationships, accessible communication, flexibility and safe and inclusive spaces, support involvement. Conversely, power imbalances, lack of diverse perspectives, logistical and ethical complexities and the time and resources required hinder involvement [12, 20, 23]. Barriers that are particularly relevant to diverse and under-represented young people in mental health research are often not specified in the literature, but the little research that does exist identifies certain factors as relevant to particular groups [20]. For example, young people from lower socio-economic groups can be excluded from digital involvement opportunities [24] and scheduling involvement activities can be challenging for young people with disabilities or health conditions [25]. Mistrust of researchers can discourage ethnically diverse young people from involvement [26, 27] and hesitancy in speaking in front of adults is reported as a barrier for refugee youth [28]. Further, barriers and facilitators are often reported from researchers’ perspectives, with little known about the views of young people [20, 29]. Whilst many of these reported barriers could be applicable to young people’s involvement in research more generally, certain barriers are particularly relevant to mental health research. These include stigma and identity, researcher concerns about risks and safety and lack of youth trust in mental health services [20, 30]. To the best of our knowledge, no studies have explored under-represented young people’s views on barriers and facilitators to their involvement in the mental health research process, particularly among those with no prior research involvement experience. Understanding these perspectives is essential to identifying any unique barriers faced by these groups. This study, which involved youth advisors and co-researchers throughout, aims to address this gap by applying the Behaviour Change Wheel (BCW) [31] to focus groups conducted with young participants. Using this theoretical framework, “involvement” is framed as a behaviour shaped by specific individual level drivers that can be optimised through targeted strategies to promote inclusive research. Applying theory is important in advancing science and developing interventions [32] and the BCW is well established in its application to young people’s mental health [20, 33, 34] and to equity, diversity and inclusion [20, 35].

## The Behaviour Change Wheel

**Fig. 1:**
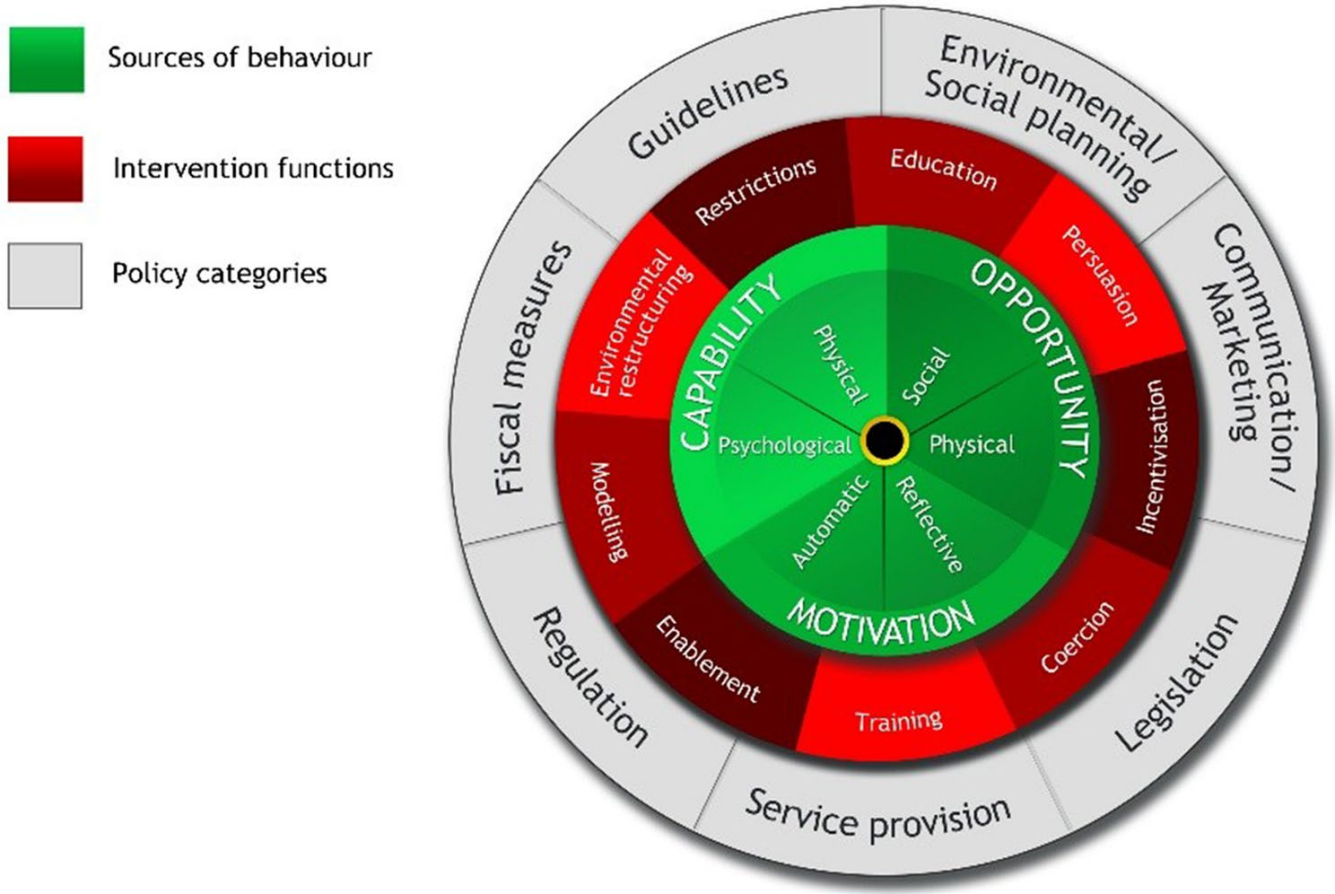
The Behaviour Change Wheel (BCW) [31]

The BCW (Fig. 1) is an integrated framework, drawn together with international behaviour change experts, from nineteen existing frameworks of behaviour change [31]. Its outer rings consist of nine intervention functions (such as persuasion and modelling) and seven policy categories (such as service provision and legislation) which can be employed to overcome barriers and enhance enablers to change. In the centre of the wheel sits the Capability-Opportunity-Motivation-Behaviour (COM-B) model of behaviour, made up of six components which interact to influence behaviour. Capability can be Physical, for example physical strength, or Psychological, such as behavioural regulation. Opportunity can be Social, for example social norms, or Physical, such as resources. Finally, Motivation can be Reflective, i.e. conscious thought processes, or Automatic, such as emotional responses. Barriers and enablers are categorised into these six components and then linked, using evidence and expert consensus, to the intervention functions and policy categories within the BCW that are most likely to effect change [36].

Further, the BCW integrates with the Behaviour Change Technique (BCT) Taxonomy (BCTTv1) [37]. BCTs represent the smallest level of ‘active ingredient’ that can be added to a behaviour change intervention, with each of 93 BCTs linked to intervention functions within the BCW [38].

### The present study

This qualitative study utilises the BCW to categorise barriers and enablers to diverse young people’s involvement in mental health research and to link these to the strategies that evidence suggests will be the most likely to bring about change. The research questions are:

1. What are the COM-B categorised barriers and enablers to diverse young people’s involvement in mental health research?
2. What intervention functions and behaviour change techniques (BCTs), linked to the BCW, can be proposed to overcome the identified barriers?

## Methods

### Participants

Young people were recruited through schools, two youth participation projects within a local authority in South-East England and through the McPin Foundation (a UK mental health research charity) Youth Network, with adverts circulated by teachers, local authority youth participation leads and McPin’s public involvement co-ordinator. Young people expressed their interest to their youth leader, teacher or to the researcher directly, in the case of McPin, and subsequently received the participation sheet and consent forms. Eligibility criteria were being between 16 and 25 years of age, being UK-based and having sufficient English proficiency to participate, with an emphasis on recruiting under-represented youth from different ethnic backgrounds, those with disabilities, people from lower-income households, migrants, refugees, or asylum seekers and those whose first language is not English. This was to capture young people able to consent to research participation who are known to be from under-represented groups.

Five focus groups were held over 6 months and included 18 young people in total. This sample size is in line with recommended numbers of focus groups to achieve saturation [39]. Participation involved completing a consent form and demographic questionnaire followed by attendance at a one-hour focus group. Materials and focus groups were completed online (via Zoom) in three instances with the remaining two groups held in person (on school or local authority premises), based on the groups’ preferences. The demographic questionnaire was informed by the Minimal Diversity Data Set [40] but adapted based on feedback from young people. All participant materials were reviewed and adapted through consultation with a Young Researchers’ Oversight Group (YROG) to ensure accessibility and sensitivity. The participant information sheet included details of the study as well as signposting information for mental health services. Young people were asked (either directly as part of the recruitment process or via their teacher/youth leader) whether there were any ways in which their participation in the focus group could be supported and accessibility arrangements made. The focus group protocol included safeguarding and confidentiality arrangements.

### Procedure

The study was approved by the university Ethics Committee (20293/003). Participants were thanked with a £25 voucher, in line with recommended NIHR rates [41], and a letter of thanks on university headed paper. For groups including participants with no prior involvement experience, questions were hypothetical and for groups with prior involvement experience, questions were based on that experience. Focus groups started with an introduction including an explanation, with examples, of what involvement in mental health research meant in the context of the research study, as well as session guidelines such as: there being no expectation of participants to discuss their own mental health, listening and respecting others’ contributions and the importance of keeping others’ contributions confidential. Questions were open-ended and mainly based on the COM-B model, with additional general questions to capture broader views from participants, ensuring that questions were guided, but not limited, by the underpinning theoretical framework [42]. Example questions for focus groups of participants who had no previous involvement experience are provided in Table 1, these were adapted for those with experience. For example, rather than asking “Where might you look if you wanted to get involved in mental health research?” these participants were asked “Where do you look if you want to…”. All participants were given the opportunity to respond to each question.

Within each question area participants were also asked whether they felt any aspects of a young person’s identity influenced this area. Focus groups were recorded, transcribed and transcripts pseudo-anonymised before recordings were deleted.

**Table 1:** Examples of focus group questions

COM-B component	Sample questions
Warm up question	Which groups/characteristics of young people (if any) do you think are under-represented in mental health research?
Psychological and Physical Capability	What skills and knowledge do you think are needed to get involved in mental health research?
Reflective Motivation	What would the benefits be of getting involved? Can you think of any downsides?
Automatic Motivation	What sorts of feelings do you think you’d experience about being involved, before during and after?
Physical Opportunity	Where might you look if you wanted to get involved in mental health research?
Social Opportunity	How would other people influence your involvement, either positively or negatively?
General questions	In what ways, if any, do you feel your identify would affect you getting involved?

Note: these sample questions use the hypothetical framing for participants with no prior involvement experience

### Data analysis

Thematic analysis was conducted (by RP) [43]. First, familiarisation with the data was achieved through repeated readings, and cleaning, of the focus group transcripts. The data were then segmented into meaningful units and first coded deductively according to the COM-B model [36]. Secondary coding of the first focus group was undertaken independently by a young co-researcher (AL), resulting in an initial agreement rate of 88%. Discrepancies were reviewed collaboratively and resolved by consensus. The remainder of the transcripts was then analysed (by RP). Secondary inductive coding was carried out (by RP) to identify more specific, data driven themes, which were defined and named. Their relevance to specific participant demographics was explored. Themes were sense-checked by AL. Following this, identified barriers (and those influences serving as both barriers and enablers) were mapped onto intervention functions using the Behaviour Change Wheel [36]. Suitable Behaviour Change Techniques (BCTs) were then selected, guided by established links from the literature [44]. Examples of operationalised BCTs, grounded in relevant literature and supplemented with suggestions offered by participants in the focus groups, are provided in Table 3. Selection took account of acceptability, practicability, effectiveness and cost-effectiveness, affordability, potential unintended consequences, and equity, using a standardised framework for evaluating interventions [36].

### Young people’s involvement

Young people were consulted in an advisory capacity throughout the study via the YROG. This was a diverse group of five young people, from within an existing advisory group, who responded to an expression of interest and have been involved in several studies within this research programme. YROG members were between the ages of 18 and 20 years. Two identified as male and three as female. White, Asian British, Arab and ‘multiple ethnic groups’ were represented. World views included Christian, Muslim and Atheist or Agnostic. Sexual orientations included heterosexual, gay and questioning. Levels of parental education ranged from GCSE to Doctoral degree (or equivalents) and the young people were all in full-time education. All five were born in the UK, but four had a parent born outside the UK and for one, English was not their first language. The group included young people who had experienced mental health problems, disability or long-term health conditions and who were neurodiverse. The young people lived in a variety of locations – urban, suburban and rural.

The YROG met roughly every three months to advise on the project. They discussed and endorsed the research questions and provided their feedback on study plans, resulting in changes to study design (such as using focus groups rather than interviews and including more participants without prior involvement experience). Consultation was via Zoom meetings and electronically through the young people commenting on documents. Meetings were held in the evenings to suit young people’s schedules and were support by a Young People’s Advisory Group co-ordinator for safeguarding reasons. One group member reviewed all participant information and as a result language was made more accessible and documents shortened. Another member of the group (AL) was a co-researcher, carrying out a deductive thematic analysis of one focus group and reviewing final themes for accuracy. The YROG reviewed and commented on the study findings and contributed to dissemination plans. The young people’s involvement was based on levels of patient and public engagement activities taken from an existing framework for youth involvement in mental health research [18], and reported using the GRIPP2 checklist for the transparent and consistent reporting of patient and public involvement activities in research [45] (see Supplementary Materials).

## Results

Summary demographics for the five focus group participants are provided in Table 2, ensuring that no participants are identifiable. The majority of participants had no prior involvement in mental health research; the two focus groups advertised through McPin included participants who were not known to each other and who had previous experience of involvement, young people within each one of the remaining three groups were known to each other and had no prior involvement experience.

**Table 2:** Summary participant demographics

Demographic	Category	Number
Gender	Male/Man/Boy	8
	Female/Woman/Girl	6
	Cis	2
	Unsure	1
	Prefer not to answer	1
Ethnicity	White British, English and Irish	4
	Black British	1
	Black African	3
	South African	1
	Mixed Kurdish	1
	British Indian	1
	Indian	1
	British Pakistani	2
	White Polish	2
Years of age	16	4
	17	5
	18	2
	19	1
	20	0
	21	0
	22	1
	23	3
	24	1
	Prefer not to answer	1
Parental qualification level	School level qualification (GCSE, A-level)	7
	Higher education qualification (degree or doctorate)	7
	Professional qualification	1
	Unknown	2
	Prefer not to say	1
Education, training, employment status	Studying for A-levels	8
	At college	1
	Moving to university	2
	At university	2
	Apprenticeship/work-based training	2
	Working – part time	2
	Not in education, employment or training	1
Caring responsibilities	Yes	3
	No	15
Sexual orientation	Heterosexual	10
	Gay/Lesbian	3
	Bi-sexual	2
	Omnisexual	1
	Prefer not to say	1
	Don’t know	1
Family financial situation	Basic expenses just met	2
	Basic expenses met with a little left over	5
	Basic expenses met with a lot left over	7
	Prefer not to say	1
	Don’t know	3
Religion/world view	Christian	5
	Muslim	3
	Hindu	1
	Atheist/Agnostic/Pagan	7
	Prefer not to answer	2
Country of Birth	UK	13
	Other – 2 Poland, 1 South Africa, 1 India, 1 Somalia	5
Native Language	English	12
	Other – 1 Zulu, 2 Polish, 1 Urdu, 1 Telugu, 1 not specified	6
Disability/health condition	Yes	10
	No	8

**Fig. 2:**
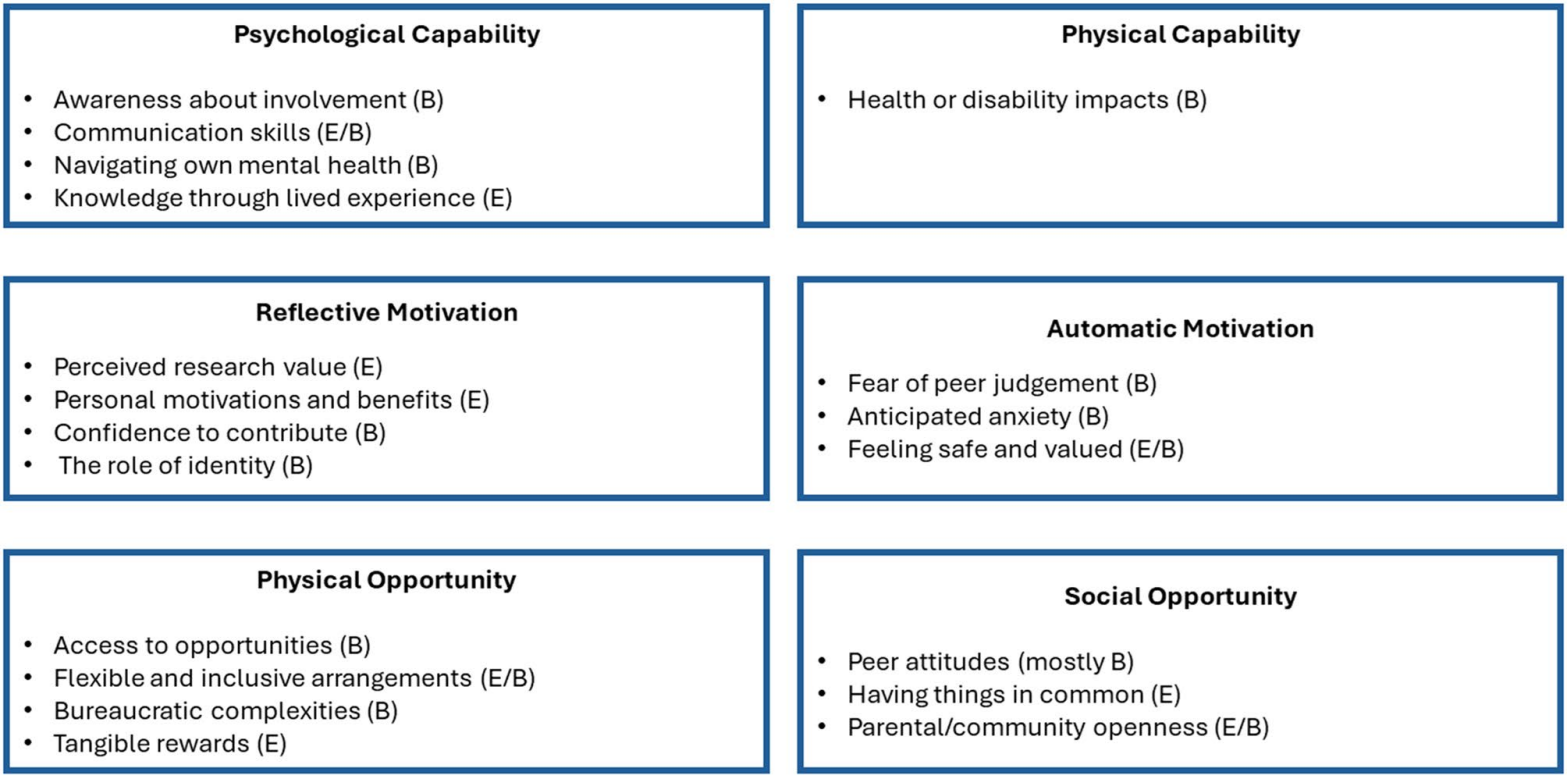
Themes categorised according to the COM-B Model. Note: (E) refers to enablers, (B) refers to barriers and (E/B) refers to themes which were both barrier and enabler

Overall 19 themes were identified covering all six COM-B domains (Fig. 2). Four themes were aligned with each of Psychological Capability, Reflective Motivation and Physical Opportunity, three to Social Opportunity and Automatic Motivation and one to Physical Capability. Five themes were enablers, ten were barriers and the remaining four themes were both enablers and barriers (depending on the particular aspect of the theme and/or the identity of the participant). Themes are described in detail below highlighting participants’ quotes. Where themes were reported as particularly pertinent to a particular identity or characteristic, these are referenced.

To preserve anonymity, participant identifiers were allocated, where a quote from participant 1 in focus group 1 is indicated by FG1P1, a quote from participant 2 in focus group 5 is FG5P2 etc.

### Psychological Capability

#### Awareness about involvement

Limited awareness and understanding of involvement was found to be a significant barrier, particularly among participants with no prior experience of involvement. Many did not understand what involvement was, and mistook it for research participation. For example, FG5P2 thought it was “not treating the participants as just subjects, and that they’re actual people” with FG1P1 believing it was about providing “an inside view on young people’s mental health.” This uncertainty extended to where such opportunities could be found. With one participant, FG1P2 acknowledging that they had “Genuinely no idea” and another, FG5P1 speculated that “I’d probably start with looking online and… at university websites.” Some participants attributed this lack of awareness to their socio-economic context. FG1P1 noted that “I’ve never heard about it and I think in other wealthier schools, they would have talked about it.” Even those with prior experience described finding opportunities by chance, as FG2P1 explained “I only found out about…it through word of mouth, it wasn’t something that was advertised to me.” Whilst some, such as FG2P2, suggested places they would look for opportunities, including “social media posts like the Twitter, Facebook and the rest of them”, these insights highlight two core barriers within this theme. The first being confusion about what involvement means and secondly, limited knowledge of opportunities, especially for those outside well-connected networks.

#### Communication skills

One of the few skills that participants felt were necessary for involvement was the ability and confidence to communicate effectively with others. For example FG2P3 stated “I think the whole communication skill is just one thing I think of, and the rest is it’s just you being yourself.” Several aspects of communication were described by participants, including FG3P1 who explained the need to be “comfortable with this digital skill”, further noting that “you have to be good with reading and writing” as input such as reviewing documents and providing written feedback was required, outside of meetings. Whilst some participants felt confident in their communication abilities, for certain groups this was a challenge. Such groups were identified as young people for whom English is not their first language, as well as neurodiverse young people. For example, FG3P2 shared that “I’m autistic, and sometimes I struggle with language, and it being quite complicated for me.” Although most young people felt that few skills were needed above being themselves, the expectations of a certain level of communication and language abilities was highlighted as potentially challenging for some young people.

#### Navigating own mental health

There were two aspects to this theme which posed challenges for participants, particularly those who had direct personal experience of mental health issues. The first was the mental health awareness and literacy required in a mental health research project. Participants felt that a lack of this could make it difficult to engage. FG4P1 explained “a lot of young people who, when talking about their mental health, struggle with, like, defining it and talking about it, and…they’re going to struggle to get involved in a study where you have to hear about their mental health.” On top of this, participants considered that young people needed to be in the right place emotionally to get involved. FG2P1 suggested the need for “a certain level of emotional regulation…where they are actually able to engage in the research without necessarily, the entire thing being triggering.” These reflections highlight both understanding mental health and emotional preparedness as potential barriers, particularly for those with previous mental health challenges.

#### Knowledge through lived experience

Participants typically believed that involvement required recent direct personal experience of mental health issues. This knowledge was mentioned by some participants as important in allowing meaningful contributions to be made. FG3P1 explained this as meaning, “you have something to offer… experience to share…insight that’s related to the project.” This expectation was perceived as a threshold for involvement. As one participant, FG2P4, reflected “the further I get away from that [experience], I think, well, have I put too much of this behind me, to the extent where I’m a bit less relatable.” Hence whilst lived-experience was viewed as an enabler by participants, it is possible that these reflections could suggest that lack of experience (or recent experience) may inadvertently discourage some young people from putting themselves forward.

### Physical Capability

#### Health or disability impacts

Participants recognised that young people with disabilities or long-term health conditions may face additional barriers to involvement. Those with personal experience discussed how fluctuating health could impact their involvement. FG2P1 shared that they had “quite a lot of physical health conditions and…wouldn’t take part in some research right in the middle of a flare up.” FG4P2 found that having ADHD meant that distractions were hard to manage and considered that, for young people with disabilities “if you do it…in their downtime, when they need that downtime to recharge… people will be much less able to kind of engage with the study.” Thus although health related challenges may not prevent involvement altogether, it may influence or even limit how young people can take part.

### Reflective Motivation

#### Perceived research value

A key enabler of involvement, across identities, was the perceived value of the research project. Participants often viewed this value in terms of its potential to create real-world change, such as improving services. As FG5P1 reflected “knowing that it can be used by services and by professionals…to help change things or make it easier for young people to have better experiences with services in schools. It’s quite a big motivator.” Relevance was also important, as explained by FG4P3 “It needs to be something that like young people will look at and be like, yeah, maybe this is a problem. Maybe I can share something about it.” These insights suggest that young people are motivated to get involved with research when it is directly relatable to them and they can see the potential tangible impact that it will have.

#### Personal motivations and benefits

A wide range of potential personal benefits were perceived by participants, beyond just financial incentives. Foremost the opportunity for development was an important driver for some participants, as FG1P4 put it, it “create[s] more personal growth.” This could be through gaining valued experience, for example in “talking with others” as described by FG3P3, or through the potential for more formal development opportunities as for FG2P3 “to get training is an added bonus for me…something that gets to be with you for life.” For young people who were about to embark on their future careers this could be particularly valuable for their CVs or personal statements. For other young people the reward was more altruistic or intrinsic. FG3P2 described “it makes me feel like I can kind of use quite challenging experiences in my life to make a positive difference. That’s actually been really important for, like, my recovery”. Involvement could help “understand yourself” as FG1P4 put it and as FG1P1 explained “be less judgemental towards others.” Thus whilst young people’s motivators varied, the personal gains from involvement were recognised as a strong enabler, both by young people with prior involvement who had experienced these benefits and for those without experience who could envisage them.

#### Confidence to contribute

Many participants, including those with prior involvement experience, had low levels of self-efficacy in their ability to make a valuable contribution. This often stemmed from comparisons with others. Some worried that they were “not smart enough to contribute anything useful” as FG1P1 explained, especially if they were put in a group with peers from perceived more academic schools. This was a sentiment shared by FG2P3 who explained feeling “kind of inferior… like anything you’re about to say is maybe not too good.” Others, including FG2P4, wanted to be sure that they had value to add “I wouldn’t say I ever have, like, imposter syndrome or anything, but I do always feel like I have to make sure that I’m, you know, really the right person for this study.” In this theme young people’s belief in their capabilities was shaped partly by social influences and the fear of judgement described below.

#### The role of identity

A number of aspects of identity were mentioned as a barrier to involvement, including gender, ethnicity and care experience. Several male participants, such as FG1P5, described how masculine norms discouraged involvement, “a lot of [men/boys] would think it’s a waste of time because they just think people should suck it up.” Another male participant, FG5P2 described “toxic masculinity, in that you’re meant to display as strong and showing no emotion” and that “talking about mental health…makes them feel weak.” Some participants noted that, within some ethnic minority communities, mental health was stigmatised. Reflecting on their own experience of being Asian, FG4P4 explained that this was because “Asian parents, in my experience, don’t really believe in the idea of mental health.” Being care experienced was also mentioned by FG2P1, who described the feeling of being “so other” leading to a belief that “shaping the way research is done, is not even something you would consider”. For these groups identity-based norms led to a belief that involvement in mental health research was not for them.

### Automatic Motivation

#### Fear of peer judgement

One of the most striking themes, particularly for those with no previous involvement experience, was a feeling of fear about being judged, particularly by peers. For example FG5P2 explained the pressure not to “give like a bad answer…some opinion that only you have and that doesn’t fit the views of others,” which was tied to self-doubt and feelings of not being good enough. This was reinforced by FG1P1 who saw age as an important factor noting that “people our age [16–17 years old] are judgmental towards each other” making young people worry about appearing rude, overly friendly or just not aligned with others. This participant went on to explain that the worry was heightened by the proliferation of social media where things said in a meeting may be spread more widely “nowadays everything and anything goes viral”. These fears were not only related to speaking up in front of peers but extended to, for example, authoring a blog that other young people might read and find offensive or disagree with. Young people’s sensitivity to opinions of their peers could negatively affect involvement, especially in a modern context where these influences are amplified.

#### Anticipated anxiety

Participants described other general feelings of being worried or anxious in anticipation of involvement, especially when this would be a new experience. For some this was linked to discomfort with unfamiliar situations. For example, for FG1P3 who had not been involved before, this was a barrier, “I don’t really like meeting new people…that kind of would hold me back a bit.” In addition, one participant, FG3P2 remembered their first experience of involvement which was during a period of not being in education, employment or training, “It was the first thing I’d done in like, over a year. So I was, I was terrified of everything, the social aspect of doing things, like writing emails, my social anxiety was just through the roof”. These accounts demonstrate how, what might be seen as simple tasks, can feel overwhelming, especially for those who have been socially isolated.

#### Feeling safe and valued

Several participants described the importance of feeling safe and welcomed in an involvement environment. These feelings were closely linked to confidentiality, with FG2P3 noting, “I am more.. I’m open if I know that it’s completely anonymous. I could actually say everything, and I’ll feel safe about it.” Online formats (such as Zoom) could foster this sense of safety, FG5P2 explained that these were “much more appealing because you’re in your own space, and you feel more safe and open to talk about stuff.” Conversely, environments that were perceived as non-inclusive could make participants feel unwelcome, especially for LGBTQIA+ young people. FG4P2 gave examples of such situations which could include those which were “unaccepting of my needs or like pronouns, basically feeling unwelcome or like my input may not necessarily be fully accepted.” Neurodiverse participants such as FG3P2 echoed similar concerns, stating that “it’s harder…to get involved in research when you’re worried about being treated not very nicely”. Although most young people described experiencing welcoming environments, a small number warned of possible barriers to those with certain identities.

### Physical Opportunity

#### Access to opportunities

Participants without prior involvement perceived access to opportunities as limited, particularly in less resourceful environments. For example, FG1P1 highlighted that “lower income families may not be well represented… this is the first time that we’ve ever had a focus group in our school” and another participant in the same group expressed disappointment at the lack of outreach from local universities. Others, who had never seen opportunities advertised, perceived structural barriers for “people who are disabled or people who are home-schooled, or people who live in locations where there’s not enough transport” (FG4P3). Those who had previous experience described how access depended on being in certain networks which were not widely available or representative. For example FG3P2 was “signed up to like, certain newsletters and or social media sites… otherwise I wouldn’t see them.” For all participants visibility and access to opportunities was lacking, especially if researchers were committed to addressing issues of under-representation.

#### Flexible and inclusive arrangements

Almost all participants strongly emphasised the importance of flexibility and inclusivity in the structure of involvement. This included induction processes, with the opportunity to “get into the open what all of our fears were” suggested by FG3P2, flexible scheduling and accessible formats, timings, and channels, as well as the presence of an inclusive researcher (who FG4P2 suggested could come in “colourful stuff, and just like being energetic”). Views varied on whether in-person or online format was preferable, one participant, FG4P4 suggested “it’d be good if there was like, an element of like, hybrid sort of thing, where people can opt for either online or in person.” Digital exclusion was not necessarily seen as a barrier because as FG5P2 noted “you can also use your phone as well to get on a call.” Ensuring venues were accessible for disabled people was an enabler, as well as timings suited to young people’s schedules as FG2P2 considered “morning, afternoon hours, people are busy with work, schools and stuff like that. So evening hours and also maybe holiday seasons and stuff like that” were suggested (excluding key exam periods). Overall participants suggested being led by young people and, as FG5P1 put it “finding out [what] the majority would prefer, making sure you can have as many people as possible being happy.”

#### Bureaucratic complexities

Lengthy and complex processes were highlighted as hard to understand and off-putting. One participant with extensive involvement experience explained “The criteria for getting involved is very strict and very narrow.” (FG3P2). It was suggested by FG5P2 that “making [forms], very simple, like yes or no questions even, so, it doesn’t really matter who you are, you’d still be able to do it” would help. For one care-experienced participant FG2P1, the assumptions embedded in the application process felt exclusionary, particularly when it came to requests for personal information: “I was a little bit uncomfortable about giving like a contact, also, because they were expecting like a family member or something, and I don’t have any family.”

#### Tangible rewards

There was unanimous agreement that payment for time spent on involvement opportunities was an enabler. This was because, as several participants highlighted, many young people experience financial insecurity. FG2P1 elaborated “I have to be quite wise with my time. And it’s like, I can’t really afford to do stuff for free.” FG2P4 added that as a result “having any sort of paid incentive might be the thing that actually gets them over that kind of otherwise barrier.” However, flexibility in the type of payment was also seen as important, as suggested by FG3P2 “giving young people the choice of what they want, in terms of, do they want to be paid with vouchers? Do they want to be paid by bank transfer…because some young people… it might mess with things like benefits.” Other forms of benefit such as receiving training or developing experience and skills to add to your CV and aid future career were also identified as highly valued, in addition to, but not as a substitute for, financial recompense. As FG5P2 explained, “thinking of a future career is really important right now. Yeah, I think gaining the skills of like analysis, research and presenting data would be important aspects to think of”.

### Social Opportunity

#### Peer attitudes

The views of other young people in participants’ networks were consistently perceived as a barrier to getting involved. FG1P1 anticipated their peers’ response to be, ““why would you want to do that? It’s a waste of time”. It would make someone reflect on why they are doing it.” There was a reluctance, amongst some participants, to discuss any issues related to mental health with peers, especially those in their own social circle “I don’t speak about this a lot with some of some of my friends from school, because… I’m going to get laughed at.” FG4P4 suggested that being in a group where participants did not know each other could allay that concern “maybe you prefer you didn’t know anyone, and you can be a bit more open that way.” Overall peer attitudes were a powerful influence that generally would put young people off engaging with involvement activities.

#### Having things in common

Participants emphasised the value of shared experience in involvement. This was particularly salient for young people from less well represented groups. FG2P2 described “feel[ing] more comfortable participating in a research study that kind of focus on you know, people of the same category as them … people that identify as LGBTQIA+.” FG4P4 noted that “Having a wide variety of individuals present” would mean that “no one feels like the odd one out” and FG3P2 agreed adding that one of the benefits of their involvement had been “meeting other people who are similar to me and had the same experiences.”

#### Parental/community openness

Participants highlighted the influential role of parents and families in shaping considerations about involvement. Middle Eastern and Muslim cultures were identified by participants as traditional and conservative where issues around mental health were described by FG1P2 as “brushed under the carpet” which would put young people off getting involved. As FG2P1 explained, they worried that families would assume “there’s something wrong with you”. This was thought to be a particular concern for young people from immigrant families who would feel pressure to appear happy to their families. On the other hand, a small number of participants found their parents to be enablers. FG5P1 described their parents as being “very good, they’re very supportive” but acknowledged that “it did take some time to get to that point. So there would have been a time where it would have been more difficult for me.”

### Behaviour Change Techniques, intervention functions and young people’s suggestions

Each barrier, along with themes that acted as both barriers and enablers, is mapped to a corresponding intervention function and BCT in Table 3. Evidence-based examples from the literature, incorporating ideas spontaneously offered by participants in the focus groups, illustrate how these can be operationalised.

**Table 3:** COM-B categorised themes mapped to intervention functions and BCTs

COM-B	Theme	Intervention functions	BCTs	Illustrative example
Psychological Capability	Awareness about involvement	Education	Instruction on how to perform behaviour	School or college outreach programme targeting under-represented students and schools in less affluent areas to work in teams with academic researchers on research projects [46]
	Communication Skills	Enablement	Social support	Peer-led workshops, induction sessions and ice-breaker activities to support contributions [47]
	Navigating own mental health	Enablement	Social support Restructuring the physical or social environment	Creating individual well-being plans with young people to support their needs [48] Providing content warnings in advance so young people know what to expect, have quiet rooms or spaces available in case young people need to take a break [48]
Physical Capability	Health or disability impacts	Enablement	Restructuring the physical environment Social support	Holding involvement activities in accessible venues, ensuring regular breaks [49] and quiet spaces. Providing catch up opportunities for young people who miss sessions for health reasons [25]
Reflective Motivation	Confidence to contribute	Persuasion	Behavioural practice/rehearsal Verbal persuasion about capability	Capacity building programmes for young people in preparation for an involvement opportunity [50]
	The role of identity	Education and Persuasion	Credible source Social comparison	Identify relevant social media influencers to partner with to promote involvement [51]. For example as an ambassador, as illustrated here: https://www.psychreg.org/mix-charity-announces-race-across-world-winner-new-ambassador/
Automatic Motivation	Fear of peer judgement	Persuasion	Reduce negative emotions	Consider options for confidentiality and anonymity of the young people involved [52] – and discuss preferences with the young people, explaining the advantages and disadvantages of different options.
	Anticipated anxiety	Enablement	Reduce negative emotions	Ensure facilitators are approachable and agree “rules of engagement” with young people in advance, including emphasising the optional nature of involvement [25]. Using creative techniques to support contributions such as think-pair-share and reduce task related anxiety [53]
	Feeling safe and valued	Enablement	Reduce negative emotions	Using affirmative and empowering facilitation to ensure all young people feel included and heard [54]
Physical Opportunity	Access to opportunities	Environmental restructuring	Restructuring the physical environment Prompts/cues	Proactive outreach to young people in different locations e.g. through social media but also physically in schools, GP surgeries, youth groups, job centres etc. [55]
	Flexible and inclusive arrangements	Environmental restructuring	Restructuring the physical environment	Consult young people about timings and mode of meetings, being open to holding meetings in evenings, weekends or school holidays. Offer choice of online or in accessible locations, on public transport routes [56]. Make efficient use of young people’s time, offering different levels of commitment.
	Bureaucratic complexities	Environmental restructuring	Restructuring the physical environment	Avoid lengthy forms and jargon, offer options for providing information in formats other than written e.g. audio recording, call [55]
Social Opportunity	Peer attitudes	Modelling	Social comparison Information about others’ approval	Create (or identify existing) videos (for example https://youtu.be/4-C6ytXbSNE) or blogs (such as https://arcwestarchive.org.uk/news/involving-young-people-in-mental-health-research-a-young-persons-perspective/) showing young people already involved in mental health research, to be shared with young people when recruiting to an involvement opportunity
	Parental/community openness	Enablement/Environmental Restructuring	Credible source Social support	Partner with relevant cultural organisations and communities (such as Taraki - Mental Health in Punjabi Communities) to promote involvement and mental health research

### Reflections of the Young Researchers’ Oversight Group

The themes identified in this study resonated with our YROG members and reflect our own experiences of involvement. In many cases they mirrored the focus group findings that awareness of, and access to, involvement opportunities were often shaped by “affluence” and privilege. We felt this was partly due to the kinds of connections that come from, for example, having a family member in a professional or senior role within academia or healthcare – someone who could introduce a young person to involvement groups or provide access to opportunities. The group linked this to “cultural capital”, in other words, who and what you know. We also agreed that parental attitudes to mental health could act as a barrier, especially in some communities (including Arab, Middle Eastern and Muslim communities), where mental health was more stigmatised. We discussed the difference between these dynamics compared with the “western norm to talk about mental health” and how this leads to under-representation. Communication and language skills were another barrier we had too experienced. We also highlighted the complex jargon often used by researchers, describing it as “overwhelming and off-putting”, especially for younger age groups. One member, having experienced mental health issues themselves, acknowledged that a fear of peer judgement might be more of a barrier for some young people than others. Having faced judgement from peers as a result, they this had ultimately strengthened their resilience and made them less concerned about being judged for their involvement in mental health research. Finally, as a group, we agreed that “perceived research value” is a hugely significant factor when recruiting. Overall, we believe there will be more involvement from young people when research feels relevant and “valuable”. We advocate that more research should be driven by the issues that young people care about, and that this will help build trust in researchers.

## Discussion

Using the BCW, this study explored barriers and enablers to youth involvement in mental health research, from young people’s perspectives, with a particular focus on under-represented groups. The five focus groups included 18 young people, many from under-represented groups - both those with, and without, prior experience of involvement, offering new perspectives that address a gap in the existing evidence base.

Across the six COM-B domains, nineteen themes were identified: both barriers and enablers. Enablers included motivational influences such as the perceived relevance and importance of research topic to young people, especially in relation to their own lived-experience. Personally valued benefits and the creation of a safe, supportive environment, also encouraged involvement. Researchers can draw on these enablers to make involvement more inclusive and appealing for under-represented young people. For example by taking on the “least adult role” and putting aside the usual signs of being an adult to interact with young people more as peers [57].

Barriers, on the other hand, often related to structural or social factors, which affected young people’s feelings of fear, anxiety and confidence, especially in group situations. To address these barriers, BCTs are proposed to restructure aspects of the physical or social environment around young people as illustrated in Table 3.

Several themes echo findings from within the existing literature. Aspects of physical opportunity such as the set up of involvement activities in terms of timings, location and payment are common and have been reported in recent relevant systematic reviews of youth involvement in mental health research [12, 20]. Similarly lack of visibility and accessibility of involvement opportunities, have also been found in previous studies [58], as has the enabler of young people being made to feel safe and comfortable [12, 58]. Many barriers were common across different groups of young people and addressing them would help ensure that opportunities are accessible to all young people, regardless of background or circumstance.

The inclusion of young people without prior involvement experience in the present study, coupled with the focus on under-represented groups, has identified additional themes not widely discussed in the existing literature. For example, for participants without prior involvement experience, the term “involvement” itself was difficult to grasp. This conceptual vagueness has been critiqued in the wider health and social sciences literature, highlighting the need for researchers to explain involvement clearly, in lay terms, using practical examples [59]. To this end, proactive outreach in schools to raise awareness of research involvement has been successful in high schools for students under-represented in STEM subjects [46].Coupling this type of approach with recruiting in a wider breadth of locations to capture young people not in mainstream provision, could serve to widen access [55].

Another barrier identified in the present study, relating specifically to mental health research, was young people’s emotional readiness and ability to discuss mental health topics and manage their own mental health during involvement. This is a concern that can make researchers hesitant to involve young people in research into highly sensitive mental health topics such as self-harm and suicide [60]. Provision of quiet spaces and content warnings [48], individual wellbeing plans and clear “rules of engagement” [25] have all been used as strategies to support young people to be involved in mental health research, to address potential negative responses and build confidence.

Failure to prepare young people for their involvement could also contribute to the anxiety and lack of confidence in their ability to contribute felt by young people, particularly with respect to communication [29]. Induction activities such as ice-breakers and creative methods for idea-sharing such as pair or small group activities have been used by other researchers to address these barriers [47, 50, 53].

Physical barriers to involvement were linked with young people’s capabilities (such as health conditions or disabilities) and environmental factors, such as limited access to the networks or locations where opportunities might be advertised. Researchers need to invest time and effort into identifying widely accessible channels and mechanisms, such as using accessible local community spaces with good transport links to advertise and run involvement opportunities [55, 56], offering communication aids, providing regular breaks within sessions [49] and catch up sessions for those who are absent [25].

Peer-influence also emerged as a significant factor, particularly among young people with no prior involvement experience, who reported being less likely to engage if they feared their social circle would disapprove of them being involved in discussions about mental health, or that they would judged by other young people, for saying the wrong thing. Similar concerns have been noted in young people’s participation in school-based mental health interventions and decision-making processes [50, 61]. It is important for researchers to consider the setting where involvement opportunities will take place and ways to address possible confidentiality concerns, for example through agreeing with young people how their contributions will be credited in publications [52]. Peer role models have also been shown to be feasible and acceptable in other contexts, such as promoting physical activity among younger age groups [62]. Peer-led workshops can also make involvement more relatable while supporting youth development [47] alongside other capacity building efforts [50].

Whilst the influence of masculine ideals is well documented in terms of how it affects mental health literacy, help-seeking and mental health research involvement [20, 63, 64], participants in the present study additionally reported cultural identity as a barrier, particularly for those from Asian backgrounds, with the intersecting identities of being male and from an Asian background compounding this specific influence on mental health research involvement. To encourage involvement, evidence also suggests that modelling can be an effective intervention strategy and could involve BCTs such as providing positive social comparisons and information about others’ approval, for example using celebrities or influencers. Fostering relationships with relevant local cultural organisations to encourage participation is another strategy taken from the literature [29] aligning with the BCTs credible sources and social support.

Overall, the strategies proposed demonstrate that meaningful involvement of under-represented young people in mental health research requires researchers to go beyond traditional networks and approaches. It requires investing additional time, resources and effort into proactive outreach, creative methods, detailed preparation and ongoing support to meet young people where they are. Many of the strategies proposed here require testing and evaluation in this context. Further research is needed to implement and evaluate the effectiveness of these strategies in a real-world research context.

## Limitations

This study focused on individual level influences on young people’s involvement in mental health research rather than structural inequalities, such as racism and poverty, which affect mental health [65] and may also hinder involvement [66]. In addition, as a UK-based study, findings may not be as relevant to other countries or cultural contexts where approaches to young people’s involvement are either more or less well developed. Relatedly, although the young people participating in the study were diverse in many respects, all participants were fluent English speakers, limiting insights into language barriers. Finally, although a number of participants had no previous experience of being involved in mental health research they were, nevertheless a group of generally engaged young people, for example through their membership of local youth networks or subscriptions to mental health newsletters. Whilst significant outreach efforts were made to recruit as diverse group of young people as possible, the recruitment strategy may have meant that those who are most hard to reach, and accordingly might experience the most significant barriers, may not have been included and therefore their views are absent.

## Conclusion

This study obtained the views of a diverse group of young people across ethnicity, gender, disability, socio-economic status and, importantly, included both those with and without prior involvement experience. It was strengthened by involving young people as advisors and co-researchers throughout the research process and incorporating their feedback into the study design and analysis of findings.

The application of the BCW, a robust theoretical framework, allowed a range of barriers and enablers to be identified and categorised, spanning all domains of the COM-B model. Barriers are highlighted that were particularly relevant to both certain under-represented groups and identities of young people and those that are unique to the context of mental health research. Notable findings include a lack of understanding of what involvement is and fear of peer-judgement particularly for those without prior involvement, stigma and the negative influences of social norms for certain groups (male and Asian young people), young people’s ability to manage their own mental health and physical barriers for disabled young people and those with health conditions. The BCW, alongside suggestions from young people in the focus groups, led to recommended intervention strategies which include changes to the structure of the involvement environment, outreach to widen access, efforts to support young people taking part and role models to encourage openness to involvement. Together, these strategies provide examples which are both theoretically informed and grounded in lived-experience, providing researchers with many practical approaches that can be used to improve the inclusion of diverse young people in mental health research, including those who are traditionally under-represented. This study also adds to the literature by demonstrating the applicability of the BCW to designing inclusive involvement strategies in a mental health research context and provides guidance for researchers seeking to implement inclusive and meaningful involvement practices.

## Supplementary Information

Below is the link to the electronic supplementary material.

Supplementary Material 1

## Data Availability

The data sets generated during and/or analysed during the current study are not publicly available for ethical and sensitivity reasons. Deidentified summaries of some data may be available on reasonable request from the corresponding author.

## References

[1] NHS Digital. Mental health of children and young people in England 2021 - wave 2 follow up to the 2017 survey [Internet]. NHS Digital; 2021 [cited 2023 Sep 21]. Report No. Available from: https://digital.nhs.uk/data-and-information/publications/statistical/mental-health-of-children-and-young-people-in-england/2021-follow-up-to-the-2017-survey.

[2] NHS Digital. Mental health of children and young people in England. 2023 - wave 4 follow up to the 2017 survey [Internet]. NHS Digital; 2023 Nov. Report No. Available from: https://digital.nhs.uk/data-and-information/publications/statistical/mental-health-of-children-and-young-people-in-england/2023-wave-4-follow-up.

[3] Pryjmachuk S, Kirk S, Fraser C, Evans N, Lane R, Crooks J, et al. A collaboratively produced model of service design for children and young people with common mental health problems. BMC Health Serv Res. 2024;24(1):133. 38268003 10.1186/s12913-024-10562-7 PMC10809440

[4] Wadman R, Taylor O, Qualter P, Humphrey N, Wood M. A country that works for all children and young people: improving mental health and wellbeing with and through education settings. 2024.

[5] Aguirre Velasco A, Cruz ISS, Billings J, Jimenez M, Rowe S. What are the barriers, facilitators and interventions targeting help-seeking behaviours for common mental health problems in adolescents? A systematic review. BMC Psychiatry. 2020;20:1–22. 32527236 10.1186/s12888-020-02659-0 PMC7291482

[6] Hardy R, West H, Fisher P. Exploring attitudes towards seeking help for mental health problems among university students from racially minoritised backgrounds: a systematic review and thematic synthesis. BMC Public Health. 2025;25(1):1428. 40240964 10.1186/s12889-025-22521-w PMC12001731

[7] Barrow E, Thomas G. Exploring perceived barriers and facilitators to mental health help-seeking in adolescents: a systematic literature review. Educational Psychol Pract. 2022;38(2):173–93.

[8] Cronin TJ, Pepping CA, Halford WK, Lyons A. Mental health help-seeking and barriers to service access among lesbian, gay, and bisexual Australians. Australian Psychol. 2021;56(1):46–60.

[9] Powell K, Huxley E, Townsend ML. Mental health help seeking in young people and carers in out of home care: A systematic review. Child Youth Serv Rev. 2021;127:106088.

[10] Viksveen P, Cardenas NE, Berg SH, Salamonsen A, Game JR, Bjønness S. Adolescents’ involvement in mental health treatment and service design: a systematic review. BMC Health Serv Res. 2024;24(1):1502. 39609818 10.1186/s12913-024-11892-2 PMC11606120

[11] Hayes D, Edbrooke-Childs J, Town R, Wolpert M, Midgley N. Barriers and facilitators to shared decision making in child and youth mental health: clinician perspectives using the Theoretical Domains Framework. Eur Child Adolesc Psychiatry. 2019;28(5):655–66. 30229306 10.1007/s00787-018-1230-0 PMC6514112

[12] McCabe E, Amarbayan M, Rabi S, Mendoza J, Naqvi SF, Thapa Bajgain K, et al. Youth engagement in mental health research: a systematic review. Health Expect. 2023;26(1):30–50. 36385452 10.1111/hex.13650 PMC9854331

[13] Denford S, Holt L, Essery R, Kesten J, Cabral C, Weston D, et al. Engagement in rapid public health research among young people from underserved communities: maximising opportunities and overcoming barriers. BMC Public Health. 2024;24(1):2217. 39143472 10.1186/s12889-024-19762-6 PMC11325622

[14] Liabo K, Boddy K, Burchmore H, Cockcroft E, Britten N. Clarifying the roles of patients in research. BMJ. 2018;361. 10.1136/bmj.k1463 29636343

[15] Brett J, Staniszewska S, Mockford C, Herron-Marx S, Hughes J, Tysall C, et al. A systematic review of the impact of patient and public involvement on service users, researchers and communities. Patient-Patient-Centered Outcomes Res. 2014;7(4):387–95. 10.1007/s40271-014-0065-0 25034612

[16] Brett J, Staniszewska S, Mockford C, Herron-Marx S, Hughes J, Tysall C, et al. Mapping the impact of patient and public involvement on health and social care research: a systematic review. Health Expect. 2014;17(5):637–50. 22809132 10.1111/j.1369-7625.2012.00795.x PMC5060910

[17] Whitmore C, Mytkolli L, Mangialardi N, Maghera J, Rudick A, Shephard K, et al. Partnered Recruitment: Engaging Individuals With Lived Experience in the Recruitment of Co-Design Participants. Health Expect. 2024;27(4):e14131. 38965808 10.1111/hex.14131 PMC11224126

[18] Perowne R, Rowe S, Gutman LM. Understanding and defining young people’s involvement and under-representation in mental health research: A Delphi study. Health Expect. 2024;27(3):e14102. 38872473 10.1111/hex.14102 PMC11176595

[19] Michail M. Shining a spotlight on youth involvement in mental health research: Challenges and innovations. JCPP Adv. 2024;4(4):e12285. 39734920 10.1002/jcv2.12285 PMC11669768

[20] Perowne R, Rowe S, Lajevardi A, Bingham L, Parry E, Grey G, et al. Barriers and facilitators to the involvement of under-represented children and young people (aged 8–25) in mental health research – a systematic review. Clin Child Fam Psychol Rev. 2025 Sep;22. 10.1007/s10567-025-00544-4. 10.1007/s10567-025-00544-4 PMC12660437 40983788

[21] Briefing notes for researchers [Internet]. National Institute for Health and Care Research. 2021. Available from: https://www.nihr.ac.uk/briefing-notes-researchers-public-involvement-nhs-health-and-social-care-research.

[22] Reynolds J, Ogden M, Beresford R. Conceptualising and constructing ‘diversity’through experiences of public and patient involvement in health research. Res Involv Engagem. 2021;7:1–11. 34294162 10.1186/s40900-021-00296-9 PMC8295976

[23] Totzeck C, van der Meer AS, Christiansen H, Durlach F, Li Sanchez K, Schneider S. Systematic review: patient and public involvement of children and young people in mental health research. Clin Child Fam Psychol Rev. 2024;1–18. 10.1007/s10567-024-00470-x PMC10920437 38402358

[24] Blueprint Writing Collective. A Blueprint for Involvement: Reflections of lived experience co-researchers and academic researchers on working collaboratively. Res Involv Engagem. 2022;8(1):68. 36471372 10.1186/s40900-022-00404-3 PMC9724262

[25] Walker E, Shaw E, Nunns M, Moore D, Thompson Coon J. No evidence synthesis about me without me: Involving young people in the conduct and dissemination of a complex evidence synthesis. Health Expect. 2021;24:122–33. 32510790 10.1111/hex.13078 PMC8137485

[26] Hargrove JG. A process for change: a grounded theory investigation of participatory action research as a means for countering mental illness stigma experienced by transition-aged Black youth. University of Massachusetts Boston; 2019.

[27] Mance GA, Mendelson T, Byrd B III, Jones J, Tandon D. Utilizing community-based participatory research to adapt a mental health intervention for African American emerging adults. Progress community health partnerships: Res Educ action. 2010;4(2):131–40. 10.1353/cpr.0.0112 20543488

[28] Inge E, Pérez-Aronsson A, Ibrahim K, Aljeshy R, Sarkadi A, Warner G. Ameliorating epistemic injustice in practice: Communication strategies in a research project with refugee youth coresearchers. Health Expect. 2024;27(1):e13926. 38031659 10.1111/hex.13926 PMC10726263

[29] Warraitch A, Wacker C, Buckley E, Bourke A, Hadfield K. How to address the barriers to meaningful adolescent involvement in health research: A qualitative study. J Res Adolescence. 2024;34(4):1626–41. 10.1111/jora.13031 PMC11606261 39428699

[30] Bailey K, Allemang B, Vandermorris A, Munce S, Cleverley K, Chisholm C, et al. Benefits, barriers and recommendations for youth engagement in health research: combining evidence-based and youth perspectives. Res Involv Engagem. 2024;10(1):92. 39223602 10.1186/s40900-024-00607-w PMC11370084

[31] Michie S, Van Stralen MM, West R. The behaviour change wheel: a new method for characterising and designing behaviour change interventions. Implement Sci. 2011;6:1–12. 21513547 10.1186/1748-5908-6-42 PMC3096582

[32] Craig P, Dieppe P, Macintyre S, Michie S, Nazareth I, Petticrew M. Developing and evaluating complex interventions: the new Medical Research Council guidance. BMJ. 2008;337. 10.1136/bmj.a1655 PMC2769032 18824488

[33] Ananya A, Tuuli J, Perowne R, Gutman LM. Barriers and Facilitators to User Engagement and Moderation for Web-Based Peer Support Among Young People: Qualitative Study Using the Behavior Change Wheel Framework. JMIR Hum Factors. 2025;12(1):e64097. 39908551 10.2196/64097 PMC11840382

[34] Perowne R, Gutman LM. Barriers and enablers to the moderation of self-harm content for a young person’s online forum. J mental health. 2024;33(3):357–65. 10.1080/09638237.2022.2069721 35574666

[35] Gutman LM, Perowne R, Younas F, O’Hanrachtaigh E. Making hybrid work for diverse staff in higher education: a behaviour change approach. High Educ Q. 2023.

[36] Michie S, Atkins L, West R. The behaviour change wheel. a guide to designing interventions. 2014;1:1003–10.

[37] Michie S, Richardson M, Johnston M, Abraham C, Francis J, Hardeman W, et al. The behavior change technique taxonomy (v1) of 93 hierarchically clustered techniques: building an international consensus for the reporting of behavior change interventions. Ann Behav Med. 2013;46(1):81–95. 23512568 10.1007/s12160-013-9486-6

[38] Michie S, Atkins L, West R. The behaviour change wheel. a guide to designing interventions. 2014;1:1003–10.

[39] Guest G, Namey E, McKenna K. How many focus groups are enough? Building an evidence base for nonprobability sample sizes. Field methods. 2017;29(1):3–22.

[40] Stadler G, Chesaniuk M, Haering S, Roseman J, Straßburger VM, Martina S, et al. Diversified innovations in the health sciences: Proposal for a Diversity Minimal Item Set (DiMIS). Sustainable Chem Pharm. 2023;33:101072.

[41] Payment Guidance for Researchers and Professionals [Internet]. National Institute for Health and Care Research. 2023 Jul. Report No. Available from: https://www.nihr.ac.uk/documents/payment-guidancefor-researchers-andprofessionals/27392.

[42] McGowan LJ, Powell R, French DP. How can use of the Theoretical Domains Framework be optimized in qualitative research? A rapid systematic review. Br J Health Psychol. 2020;25(3):677–94. 32558289 10.1111/bjhp.12437

[43] Braun V, Clarke V. Using thematic analysis in psychology. Qualitative Res Psychol. 2006;3(2):77–101.

[44] Cane J, Richardson M, Johnston M, Ladha R, Michie S. From lists of behaviour change techniques (BCT s) to structured hierarchies: comparison of two methods of developing a hierarchy of BCT s. Br J Health Psychol. 2015;20(1):130–50. 24815766 10.1111/bjhp.12102

[45] Staniszewska S, Brett J, Simera I, Seers K, Mockford C, Goodlad S, et al. GRIPP2 reporting checklists: tools to improve reporting of patient and public involvement in research. BMJ. 2017;358. 10.1136/bmj.j3453 PMC5539518 28768629

[46] Cramer C, Sheetz L, Sayama H, Trunfio P, Stanley HE, Uzzo S. NetSci High: bringing network science research to high schools. In. Springer; 2015. pp. 209–18.

[47] Thomson A, Peasgood E, Robertson S. The Youth Patient and Public Involvement Café—A youth-led model for meaningful involvement with children and young people. Health Expect. 2022;25(6):2893–901. 36065124 10.1111/hex.13597 PMC9700192

[48] Webb M, Cooper C, Hemming L, Dalton A, Unity E, Simmons MB, et al. Involving young people with lived and living experience of suicide in suicide research. Crisis. 2024. 10.1027/0227-5910/a000938 PMC11370782 38353004

[49] Bailey S, Boddy K, Briscoe S, Morris C. Involving disabled children and young people as partners in research: a systematic review. Child Care Health Dev. 2015;41(4):505–14. 25323964 10.1111/cch.12197

[50] Kelleher C, Seymour M, Halpenny AM. Promoting the participation of seldom heard young people: a review of the literature on best practice principles. 2014.

[51] Wentzell K, Walker HR, Hughes AS, Vessey JA. Engaging social media influencers to recruit hard-to-reach populations. Nurs Res. 2021;70(6):455–61. 34334700 10.1097/NNR.0000000000000544 PMC8563382

[52] Cullen O, Walsh CA. A narrative review of ethical issues in participatory research with young people. Young. 2020;28(4):363–86.

[53] Farr MB, Lawford H, Hull K, Stowe S, Jensen M, Ross KT. The Impact of a Bookend Think-Pair-Share Intervention on Anxiety and Student Collaboration in a Community College Human Physiology Course. HAPS Educ. 2020;24(1):33–42.

[54] Iacono G, Holle L, Loveland E, Borel C, Horton E, Olson H, et al. Community-based participatory action research with LGBTQIA+ youth during the COVID-19 pandemic: Reflections from a collaborative autoethnography. Qualitative Social Work. 2024;23(6):970–89.

[55] Centring young people in mental health research - ensuring diversity and inclusivity [Internet]. The McPin Foundation. 2023. Available from: https://mcpin.org/resource/centring-young-people-diversity/.

[56] Mawn L, Welsh P, Stain HJ, Windebank P. Youth Speak: increasing engagement of young people in mental health research. J Mental Health. 2015;24(5):271–5. 10.3109/09638237.2014.998810 26193175

[57] Atkinson C. Ethical complexities in participatory childhood research: Rethinking the ‘least adult role’. Childhood. 2019;26(2):186–201.

[58] Watson R, Burgess L, Sellars E, Crooks J, McGowan R, Diffey J, et al. A qualitative study exploring the benefits of involving young people in mental health research. Health Expect. 2023. 10.1111/hex.13722 PMC10349260 37073725

[59] Hughes M, Duffy C. Public involvement in health and social sciences research: a concept analysis. Health Expect. 2018;21(6):1183–90. 30159960 10.1111/hex.12825 PMC6250854

[60] Wadman R, Williams AJ, Brown K, Nielsen E. Supported and valued? A survey of early career researchers’ experiences and perceptions of youth and adult involvement in mental health, self-harm and suicide research. Res Involv Engagem. 2019;5(1):16. 31164992 10.1186/s40900-019-0149-z PMC6489170

[61] Foulkes L, Andrews JL. Accounting for the adolescent social context in school mental health interventions. Eur Child Adolesc Psychiatry. 2025;34(2):803–5. 10.1007/s00787-024-02439-1. 38642115 10.1007/s00787-024-02439-1

[62] Morgan K, Van Godwin J, Cannings-John R, Hallingberg B, Moore G, Pell B, et al. A mixed-methods process evaluation of the feasibility and acceptability of involving community and peer role models within a physical activity intervention for primary-school-aged girls (the CHARMING study). BMC Public Health. 2023;23(1):1950. 37805485 10.1186/s12889-023-16826-x PMC10560422

[63] Clark LH, Hudson JL, Rapee RM, Grasby KL. Investigating the impact of masculinity on the relationship between anxiety specific mental health literacy and mental health help-seeking in adolescent males. J Anxiety Disord. 2020;76:102292. 33010663 10.1016/j.janxdis.2020.102292

[64] Sheikh A, Payne-Cook C, Lisk S, Carter B, Brown JS. Why do young men not seek help for affective mental health issues? A systematic review of perceived barriers and facilitators among adolescent boys and young men. Eur Child Adolesc Psychiatry. 2025;34(2):565–83. 39004687 10.1007/s00787-024-02520-9 PMC11868194

[65] Pinfold V, Thompson R, Lewington A, Samuel G, Jayacodi S, Jones O, et al. Public perspectives on inequality and mental health: A peer research study. Health Expect. 2024;27(1):e13868. 37786331 10.1111/hex.13868 PMC10768865

[66] Pearce C. The complexities of developing equal relationships in patient and public involvement in health research. Social Theory Health. 2021;19(4):362–79.

